# P-393. Examining Breast Surgical Site Infections: A Case-Control Study on the Effect of Skin Antisepsis Agents and Other Contributing Factors

**DOI:** 10.1093/ofid/ofae631.594

**Published:** 2025-01-29

**Authors:** Sonja Anic, Denice S Loisy, Bryan Knepper

**Affiliations:** UCHealth, Colorado Springs, Colorado; UCHealth, Colorado Springs, Colorado; UCHealth, Colorado Springs, Colorado

## Abstract

**Background:**

Postoperative surgical site infections (SSI) in breast mastectomy and reconstruction cases persist despite efforts to control extrinsic factors. SSI may lead to prolonged hospitalization, increased costs of patient and hospital, and patient hardship. Current skin antisepsis practices warrant strict observation, application, and exploration of efficacy, as Gram-positive microorganisms are most commonly associated with breast surgery. The use of an alcohol-based skin preparation has proven to be more effective than those that are aqueous-based, We evaluated potential patient and procedure risk factors for SSI, including the perioperative skin prep used in breast mastectomy, breast plastic reconstructive, and combination breast and plastic cases.

**Methods:**

A case-control study of breast procedures was performed. Procedures were identified between January 1, 2022 and December 31, 2023 from two campuses in the same health system. Procedures were divided into three case types: breast-only, plastics-only, and combination of breast and plastics. In addition to skin prep solution, factors assessed for SSI risk were: age, in-patients, out-patients, body mass index, diabetes mellitus, duration of procedure, wound class, and ASA.

**Results:**

45 SSI cases were identified, and 92 controls were randomly selected. Factors associated with SSI included duration of case, BMI, case type, inpatient procedure, emergent procedure, and wound class. Most SSIs were superficial with *Staphylococci* the most common organism isolated. Iodophor paint and/or scrub were used in the majority of cases and controls; Chloraprep was used in 0 SSI cases and 3 controls.

**Conclusion:**

Attention should be focused on the prevention of SSI due to its serious nature. Multiple factors can contribute to the development of SSI and its prevention. The incidence of SSI was higher in patients who underwent either plastics-only or a combination breast/plastics procedures; iodophor skin prep may also be an area of concern. Next steps include implementing a trial of Chloraprep skin prep for all breast procedures, continuing to evaluate other known risk factors, and investigating other potential opportunities, including postoperative patient management.

**Disclosures:**

**All Authors**: No reported disclosures

